# Influences of Growth-Related Myopathies on Peptide Patterns of In Vitro Digested Cooked Chicken Breast and Stress-Related Responses in an Intestinal Caco-2 Cell Model

**DOI:** 10.3390/foods13244042

**Published:** 2024-12-14

**Authors:** Yuwares Malila, Sunitta Saensa-ard, Chanikarn Kunyanee, Nalinrat Petpiroon, Nantanat Kosit, Sawanya Charoenlappanit, Narumon Phaonakrop, Yanee Srimarut, Sasitorn Aueviriyavit, Sittiruk Roytrakul

**Affiliations:** 1 National Center for Genetic Engineering and Biotechnology, 113 Thailand Science Park, Khlong Nueng, Khlong Luang, Pathum Thani 12120, Thailand; nantanat.kos@ncr.nstda.or.th (N.K.); sawanya.cha@ncr.nstda.or.th (S.C.); narumon.pha@biotec.or.th (N.P.); yanee.sri@biotec.or.th (Y.S.); sittiruk@biotec.or.th (S.R.); 2 National Nanotechnology Center (NANOTEC), National Science and Technology Development Agency (NSTDA), 111 Thailand Science Park, Khlong Nueng, Khlong Luang, Pathum Thani 12120, Thailand; sunitta.sae@ncr.nstda.or.th (S.S.-a.); chanikarn.kun@nanotec.or.th (C.K.); nalinrat.pet@nanotec.or.th (N.P.); sasitorn@nanotec.or.th (S.A.)

**Keywords:** chicken meat, growth-related myopathy, in vitro protein digestion, oxidative stress, digital polymerase chain reaction

## Abstract

The objective of this study was to determine the effects of growth-related myopathies, i.e., normal, wooden breast (WB), white striping (WS), and the combined lesions of WS and WB (WS + WB), on the molecular response of Caco-2 cells. A total of 24 cooked chicken breasts (*n* = 6 per myopathy) was subjected to an in vitro digestion using an enzymatic process mimicking human gastrointestinal digestion. Based on peptidomics, in vitro protein digestion of the abnormal samples, particularly WB meat, resulted in more peptides with lower molecular mass relative to those of normal samples. The cooked meat hydrolysates obtained at the end of the digestion were applied to a Caco-2 cell model for 4 h. The cell viability of treated normal and abnormal samples was not different (*p* ≥ 0.05). Absolute transcript abundances of genes associated with primary oxidative stress response, including nuclear factor erythroid 2 like 2, superoxide dismutase, and hypoxia-inducible factor 1 were determined using a droplet digital polymerase chain reaction. No significant differences in transcript abundance of those genes in Caco-2 cells were demonstrated between normal and the abnormal samples (*p* ≥ 0.05). Overall, the findings supported that, compared to normal meat, the cooked chicken meat with growth-related myopathies might be digested and absorbed to a greater extent. The cooked abnormal meat did not exert significant transcriptional impacts regarding oxidative stress on the human epithelial Caco-2 cells.

## 1. Introduction

In the past decade, the issue of growth-related myopathies, i.e., white striping (WS) and wooden breast (WB), has raised a wide concern among meat-type poultry industry. The meat abnormality was initially recognized with the visual deviation, e.g., development of white striations and hardened ridges along with hemorrhages on the meat surface [1,2,3]. However, the profound pain points of such issues were the reduced technological properties, particularly a decreased water holding capacity and the tough texture of the cooked meat [1]. The economic impacts of the growth-related myopathies were estimated to cause a USD 1 billion annual loss in the USA alone [2].

Growing scientific information has pointed out an association between growth-related myopathies and cellular oxidative stress. Such stress was induced by insufficient vascularization associated with fewer capillaries being found among the breast muscle cells [3,4,5,6]. In addition, the distance between the capillaries and the muscle cells appeared to be farther in the pectoralis major of the affected birds [5]. Such physiological changes led to a local accumulation of reactive oxygen species (ROS), contributing to redox imbalance [3,4,7,8,9]. An increase in oxidation of lipids and proteins was consistently reported in the WB meat [10,11,12]. Such physiological stress along with the phlebitis found in the affected birds [13,14] appeared to induce chronic inflammation and muscle fiber degeneration.

Upon exposure to ROS, proteins may undergo chemical modifications, leading to conformational changes, protein aggregation, or the cleavage of polypeptide backbones [15]. Although certain conformational changes can facilitate proteolytic hydrolysis, oxidative modifications coupled with molecular aggregation may hinder the target sites from proteases [15]. Such events are more likely to lead to a reduction in protein digestibility. Moreover, repetitive consumption of high-dose oxidized diets may induce oxidative stress in the gut lumen, triggering cellular inflammation [16,17,18,19]. Indeed, our previous studies, using a series of in vitro enzymatic processes mimicking human gastrointestinal digestion demonstrated that the cooked chicken meat samples affected by growth-related myopathies were more susceptible to the in vitro protein digestion [20,21]. Whether those digested cooked meat exerted cellular impacts on the gastrointestinal tract, however, was not fully understood.

An investigation of Caco-2 cell viability using an MTT assay has been widely used as a model for studying potential cytotoxic effects on human intestinal epithelial barrier [22]. In our recent study, the effects of pepsin-hydrolyzed chicken meat with or without the WB condition on the viability of Caco-2 and Vero cells were investigated [21]. The results showed that Caco-2 cells treated with pepsin-hydrolyzed WB cooked meat (at hydrolysate concentrations ranging between 1.56 to 100 μg/mL) exhibited significantly lower cell viability than those treated with non-WB samples. The potential cytotoxic effect of the WB abnormality on human intestinal epithelial cells was suggested to be associated with an increase in hydroxyl kynurenine, a pro-oxidant, in the WB samples [23].

In general, when living creatures encounter oxidative stress, their biological cascades of antioxidant processes are activated to maintain cellular redox balance. Among these, nuclear factor erythroid 2 like 2 (*NFE2L2*), superoxide dismutase (*SOD*), and hypoxia-inducible factor 1 (HIF-1) play crucial roles as primary protective mechanisms. *NFE2L2* is a transcription factor belonging to a protein family of basic leucine zipper. A previous study addressed increased NFE2L2 expression induced under high ROS and nitric oxide conditions [24]. Encoded by the *NFE2L2* gene in humans, this transcription factor regulates a wide array of antioxidant and stress-response genes, exerting critical cytoprotective effects for the cells [25,26]. As for *SOD*, *Cu/Zn SOD*, known as cytosolic *SOD1*, detoxifies intracellular superoxide anion concentrations through catalyzing the breakdown of superoxide anion into hydrogen peroxide and water [27,28,29]. Increased SOD activity was addressed in oxidative-stress induced Caco-2 cells [30,31]. As for *HIF-1*, this transcription factor functions as a master regulator of cellular and systemic homeostatic response to hypoxia. Under oxidative stress, *HIF-1* regulates metabolic adaptation to slow down the biological pathways that consume oxygen and generate ROS [32].

Given that WS-affected meat has become more frequently observed in the market [33], there is a growing possibility that affected meat is more likely to be consumed. Therefore, the objective of this study was to elucidate the effects of different growth-related myopathies (i.e., normal, WS, WB, and WS + WB) on Caco-2 cell viability. Absolute transcript abundance values of *NEF2L2*, *HIF1A*, and *SOD1* in Caco-2 cells were also analyzed. Peptidomic profiles of the in vitro digested chicken samples were compared to identify the respective proteins susceptible to the proteases. Ultimately, the findings lay a foundation underlying the effects of growth-related myopathies on the molecular oxidative stress responses in the human gastrointestinal lumen.

## 2. Materials and Methods

### 2.1. Materials, Chemicals and Reagents

All breast samples (*Pectoralis major*) were collected from one processing batch at a local slaughterhouse (Pathum Thani, Thailand). Chemicals and solvents were of analytical grade and were purchased from Carlo Erba Reagenti (Rodano, Italy), Merck (Darmstadt, Germany), and Sigma-Aldrich (St. Louis, MO, USA). The α-amylase (EC: 3.2.1.1, 100,000 U/g) was purchased from Megazyme (Wicklow, Ireland). Pepsin (EC: 3.4.23.1, ≥3200 Units/mg), pancreatin (EC: 232–468-9, 4 × USP activity), and bile salt were purchased from Sigma-Aldrich (St. Louis, MO, USA). The human epithelial colorectal adenocarcinoma cell line (Caco-2; HTB-37™) was obtained from the American-Type Culture Collection (ATCC, Manassas, VA, USA). Dulbecco’s modified Eagle’s medium (DMEM) was purchased from Merck KGaA (Darmstadt, Germany). All reagents for the droplet digital polymerase chain reaction (ddPCR) were the products of Bio-rad (Bio-Rad Laboratories, Inc., Hercules, CA, USA).

### 2.2. Samples and Sample Preparation

A total of 24 chicken breasts classified as “normal”, “moderate WS”, “moderate WB”, or “WS + WB” were collected for further analyses. Each group contained six replicates. The classification of growth-related myopathies was conducted by one trained staff member to minimize any variations. The classification criteria were based upon the study of Malila et al. [7]. Briefly, the WS samples were classified as the breasts with white lines (1.0 mm to 1.9 mm thickness) covering the meat surface, whereas the WB samples were classified based on the appearance of bulging ridges and markedly hardened areas on the skin side. The samples were individually packed in a polyethylene bag and transported under temperatures from 0 °C to 2 °C to the Food Biotechnology laboratory (BIOTEC, Pathum Thani, Thailand). Upon arrival, the meat was stored at −20 °C until further being subjected to cooking.

The cooking procedure was similar to the one previously described in the study of Srimarut et al. [20]. In brief, the meat, individually vacuum-packed in a polyethylene bag, was immersed in a water bath set at 95 °C until the internal temperature of its thickest part reached 80 °C. After cooling in an iced water bath until the core temperature cooled down to 15 °C, the cooked samples were left to rest at 4 °C for 2 h. Subsequently, the meat was ground using a household blender.

### 2.3. In Vitro Protein Digestion

The ground cooked chicken meat was proceeded for in vitro protein digestion following a previously described method [20]. The in vitro protein digestion consisted of three steps, mimicking the enzymatic reaction in the oral, gastric, and intestinal stages. The digestion began with an incubation of 2 g (dry basis) of ground meat sample with 4 mL of buffer solution (120 mM NaCl, 5 mM KCl, and 6 mM CaCl_2_, pH 6.9), containing 75 U/mL α-amylase. After a 5 min incubation at 37 °C with constant stirring (10 rpm), 8 mL of the buffer was added, followed by 6N HCl until the pH of the sample mixture reached pH 2.0. Subsequently, pepsin was added to a final concentration of 2000 U/mL. The mixture was then incubated for 60 min at 37 °C with constant stirring (10 rpm) using an IKA^®^ TRAYSTER digital (IKA Works, Inc., Staufen, Germany). The pH of the mixture was then adjusted to pH 5.0 using 1.5 M NaHCO_3_. To mimic the intestinal phase, pancreatin (100 U/mL based on trypsin) and bile salt (10 mM) were added. The final pH of the mixture was increased to pH 6.0 using 1.5 M NaHCO_3_. The pancreatic reaction was conducted for 300 min at 37 °C with constant stirring (10 rpm). Upon completion of the in vitro digestion, the enzymatic reaction was stopped by boiling for 2 min. The mixture was then centrifuged at 3000× *g* for 30 min. Subsequently, the supernatant was filtered through a 0.2 μm membrane for further analyses.

### 2.4. Peptidomic Analysis

The 0.2 μm filtrate collected from the supernatants was subjected to molecular weight cut-off (MWCO) separation using Amicon Ultra 3000 MWCO centrifugal filters (Billerica, MA, USA) to isolate peptides with a molecular weight ≤ 3000 Da, following the manufacturer’s instruction. The peptide concentration in the filtrate was quantified using the Lowry assay [34] prior to proteomic analysis. Samples were then analyzed by liquid chromatography–tandem mass spectrometry (LC-MS/MS) on an Ultimate 3000 Nano/Capillary LC System (Thermo Scientific, Oxford, UK) connected to a ZenoTOF 7600 mass spectrometer (SCIEX, Framingham, MA, USA). Peptides were separated using an Acclaim PepMap 100 column (75 µm × 5 cm), with 0.1% formic acid in water as eluent A and 80% acetonitrile with 0.1% formic acid as eluent B. A gradient of 5–55% eluent B was applied over 30 min at a flow rate of 0.30 µL/min.

In data-dependent acquisition (DDA) mode, the instrument selected the top 50 precursor ions per MS1 survey scan above a 150-cps intensity threshold for MS/MS analysis, with a dynamic exclusion of 12 s following 2 MS/MS events. MS2 spectra were collected within an m/z range of 100–1800 and a 50 ms accumulation time in the Zeno trap, using an 80 V declustering potential. The time bins were summed to 8 across channels, with a Zeno trap threshold of 150,000 cps. A 3.0 s cycle time was employed for the top 60 DDA. Spectral matching to the UniProt Gallus gallus database (downloaded 2 March 2024) was conducted using MaxQuant v2.1.4.0 with the Andromeda search engine [35,36]. Label-free quantitation parameters included up to two missed cleavages, with a mass tolerance of 0.6 Da. Searches assumed trypsin specificity, incorporating fixed cysteine carbamidomethylation and variable modifications for methionine oxidation and N-terminal acetylation. Differential peptide peaks were identified using a false discovery rate (FDR) of <0.05 and were subsequently analyzed by one-way analysis of variance (ANOVA) via MetaboAnalyst v6.0 [37].

### 2.5. Cell Viability Assay

To determine cell viability, the human epithelial colorectal adenocarcinoma cell line (Caco-2) was obtained from ATCC (HTB-37™) and seeded at a density of 25,000 cells/cm^2^ in a 24-transwell plate for 21 days. The cells with a transepithelial electrical resistance (TEER) higher than 600 Ω·cm^2^ were then treated with 100 µL of the 0.2 μm filtrate at concentrations of 2.5, 5, 10, 25, and 50% (*v*/*v*) for 4 h to mimic intestinal transit time. The solutions of 0.1% (*v*/*v*) Triton X-100 and the DMEM supplemented with 1% pen/strep were used as a positive control and a negative control, respectively. Upon the completion of the treatment, the new medium was replaced. The cells were subsequently cultured for an additional 20 h. At the end of the experiment, the cells were washed twice with 200 µL of PBS, and cell viability was determined using the MTT assay. For the MTT assay, the tissues were incubated with 600 µL of MTT solution at 1 mg/mL in the basal side for 3 h at 37 °C [38,39,40]. After the incubation, the MTT solution was removed. The formazan was dissolved with 2 mL of isopropanol in each well with constant plate shaking for 3 h. Subsequently, 100 µL of the solution was added to a 96-well plate. The absorbance was measured at 570 nm. The percentage of cell viability, expressed as a percentage, was calculated from the absorbance obtained from each treatment group relative to that of the control. Cell viability testing was performed in triplicate.

### 2.6. Absolute Transcript Abundance Analysis

To examine the oxidative stress response at the transcriptional level of Caco-2 cells, the cells were seeded at a density of 25,000 cells/cm^2^ in a 24-transwell plate for 21 days. The cells were then treated with 100 µL of the 0.2 μm filtrate obtained from the solutions of enzyme, normal, WS, WB, and WS + WB samples at 2.5% (*v*/*v*) for 6 h. The experimental cell set treated with H_2_O_2_ at 5 mM was used as a positive control whereas the cells with DMEM supplemented with 1% pen/strep were labeled as a negative control. After the treatment, cells were washed twice with 200 µL of PBS and collected using RLT buffer from the RNeasy Mini Kit (Qiagen GmbH, Hilden, Germany) for RNA extraction. A concentration of 40 ng/µL of RNA was then converted to cDNA using the High-Capacity cDNA Reverse Transcription Kit (Thermo Fisher Scientific, Vilnius, Lithuania), and the expression of genes associated with stress response was analyzed using a ddPCR.

Primers (Table 1) associated with the oxidative stress response in human cells were designed using a Primer-BLAST (https://www.ncbi.nlm.nih.gov/tools/primer-blast/ (accessed on 1 November 2023)). Primer specificity was confirmed by the presence of a single band of PCR amplicon at the corresponded molecular weight on a 2% agarose gel.

The ddPCR was performed according to the previously described method [41]. In brief, 20 µL ddPCR mixture was prepared by mixing 1X EvaGreen supermix (Bio-Rad Laboratories, Inc., Hercules, CA, USA), 0.25 µM of forward and reverse primers, cDNA template at the optimal amount, and nuclease-free water (Table 1). As for the no-template control (NTC), an equal volume of nuclease-free water was added into the reaction instead of the template. The ddPCR mixture was then converted into a water-in-oil emulsion, containing multi-thousands (12,000–20,000) of droplets, using a QX100™ droplet generator (Bio-Rad Laboratories, Inc.) following the manufacturer’s instruction. The ddPCR reaction was amplified using a T100™ thermal cycler (Bio-Rad Laboratories, Inc.) set as follows: denaturation at 95 °C for 5 min, 40 cycles of annealing and extension at 95 °C for 30 s followed by an optimal annealing temperature (Table 1) for 1 min, and droplet stabilization at 4 °C for 5 min followed by 90 °C for 5 min. The temperature ramp rate of all steps was set at 2.0 °C/min. Afterwards, the fluorescent signal intensity of the droplets was determined using a QX200 droplet reader (Bio-Rad Laboratories, Inc.) equipped with a QuantaSoft droplet reader software version 1.7.4.0917 (Bio-Rad Laboratories, Inc.). Absolute abundance, obtained from six replicates, was expressed in a unit of copy number per 20 µL reaction.

### 2.7. Statistical Analysis

Statistical analysis was performed using R package version R 4.1.1. The significance level was set at *p* < 0.05. Before determining the effects of growth-related myopathies, the assumptions of homogeneity of variance and normal distribution were tested. The dataset, following these assumptions, was analyzed using one-way ANOVA. Mean differences were subsequently analyzed using Tukey’s HSD. On the other hand, the non-parametric dataset was subjected to the Kruskal–Wallis test, followed by Dunn’s test.

## 3. Results

### 3.1. Differences in the Peptidomic Profiles of Digested Chicken Breasts

**Figure 1:**
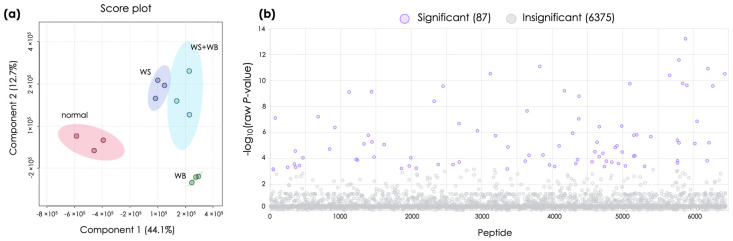
Different peptides (≤3000 Da) were released during in vitro digestion of cooked chicken breasts affected by various growth-related myopathies (normal, wooden breast (WB), white striping (WS), and WS + WB). (**a**) The scatter plot shows partial least squares discriminant analysis (PLS-DA). (**b**) Differential peptides (purple dots) are identified based on analysis of variance (FDR < 0.05).

The current study used LC-MS/MS to analyze the peptide profiles of the P5 fractions. Prior to the analysis, the hydrolysates of P5 fractions were subjected to an MW cut-off. Hence, only peptides and free amino acids with MW ≤ 3000 Da proceeded to the analysis. Overall, a total of 6462 peptides and free amino acids were detected. As depicted in Figure 1a, PLS-DA clearly distinguished between normal samples and other myopathies. The high-dimensional variations (Component 1 at 44.1%) indicated significant differences in peptides between normal and myopathic samples. Among the myopathies, the low-dimensional variations (Component 2 at 12.7%) suggested some similarities in peptide profiles among the WS, WS + WB, and WB groups. This pattern reflects the influence of growth-related myopathies on the peptides resulting from in vitro protein digestion of the cooked chicken meat.

Based on ANOVA, the peptidomic analysis revealed differential peptides among the P5 fractions of those meat samples exhibiting different growth-related myopathies (Figure 1b). Of the 6462 peptides with a molecular mass ≤ 3000 Da, 87 peptides showed differential abundance among the treatments. Heatmap analysis (Supplementary Figures S1–S5) highlighted that five peptides, which are associated with the alpha-crystallin B chain (CRYAB), fused in sarcoma/translocated in liposarcoma (FUS/TLS), cornichon family AMPA receptor auxiliary protein 3 (CNIH3), signal peptidase complex subunit 1 (SPCS1), and one uncharacterized protein, were overlapped between the WS and WS + WB groups (Supplementary Figure S4).

**Figure 2:**
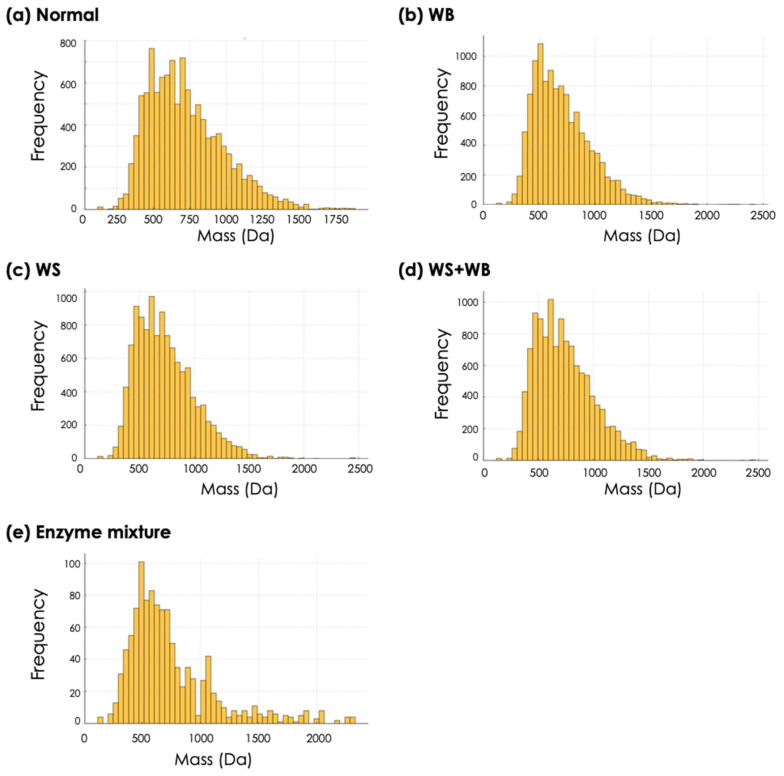
Mass distribution of ≤3000 Da peptides in the hydrolyzed cooked chicken meat. The *x*-axis represents the mass (Da), and the *y*-axis represents the frequency of the occurrence. Each histogram depicts the mass distribution within the supernatants from (**a**) normal, (**b**) wooden breast (WB), (**c**) white striping (WS), (**d**) WS + WB, and (**e**) enzyme mixture samples.

Mass distribution curves of ≤3000 Da peptides in the P5 fractions are shown in Figure 2. As for the normal samples, the majority of the peptides was also observed within the mass range of 400–800 Da with a frequency of 500–800 (Figure 2a). The larger peptides with molecular masses of 1000–1500 Da were also observed at a frequency range below 200. In comparison with that of normal samples, the mass distribution of the peptides in the P5 fractions of the WB, WS, and WS + WB samples appeared to be within a smaller MW range (Figure 2b–d). A lower frequency was observed for the peptides within a mass range at 1000–1500 Da. To assure that the results were not affected by any breakdown of the enzymes used for the in vitro digestion, a mass distribution curve of the P5 fractions containing only the enzyme mixture was also investigated (Figure 2e). The observation frequencies of each peptide with a molecular mass ≤ 3000 Da in the P5 fraction of the enzyme mixture were lower than 100 counts. The current results were consistent with the greater susceptibility of chicken meat to being affected by growth-related myopathies [20,21,42].

Considering the quantity of the peptides with MW < 1000 Da (Table 2) released during in vitro digestion of the chicken samples, the results indicated comparable percentages, ranging between 83.6–86.6%, across all samples. At this molecular size, di- and tri-peptides are considered to be small enough to be absorbed [43,44]. Therefore, the results suggested that although the normal samples were less susceptible to enzymatic hydrolysis during in vitro digestion, the numbers of low-MW peptides (<1000 Da) relative to the total peptides in the normal samples were somewhat similar to those with growth-related myopathies.

### 3.2. The Effects of Growth-Related Myopathies on Caco-2 Cell Viability

**Figure 3:**
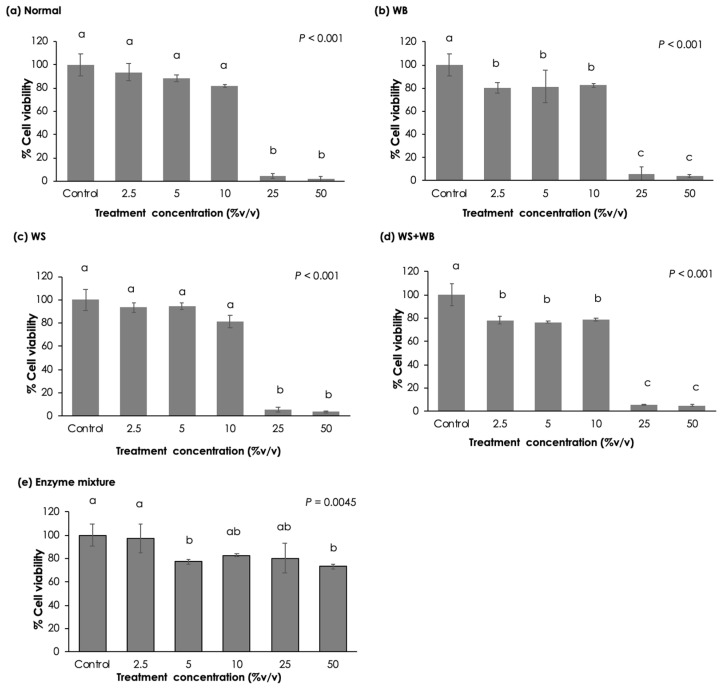
Caco-2 cell viability after treatment with 0.2 m of filtrate obtained from in vitro digested chicken breasts with different growth-related myopathies, including (**a**) normal, (**b**) wooden breast (WB), (**c**) white striping (WS), and (**d**) WS + WB samples. The filtrate obtained from enzyme mixture (**e**) was included in the experiment. Bars and error bars represent the mean and standard deviation, respectively. Different letters above bars indicate a significant difference (*p* < 0.05).

To determine potential adverse effects of the chicken meat exhibiting different growth-relayed myopathies on human gastrointestinal cell viability, Caco-2 cells were subjected to the 0.2 μm filtrates obtained from P5 fractions. As shown in Figure 3, all samples demonstrated dose-dependent effects on Caco-2 cell viability. Focusing on the normal samples (Figure 3a), the results showed no significant differences on Caco-2 cell viability at the treatment concentration from 0% to 10% (*v*/*v*) (*p* ≥ 0.05). However, the average cell viability was drastically decreased from 81.7% to 4.5% and 1.7% as the hydrolysate concentration was increased to 25% (*v*/*v*) and 50% (*v*/*v*), respectively (*p* < 0.05). A similar trend was observed when the cells were treated with the supernatant from the WS samples (Figure 3c). On the other hand, the treatments of WB (Figure 3b) and WS + WB at a concentration of 2.5% (*v*/*v*) (Figure 3d) showed lower Caco-2 cell viability values than those of their control counterparts (*p* < 0.05). No significant changes were observed as the WB and WS + WB supernatant concentration was increased between 2.5% (*v*/*v*) and 10% (*v*/*v*). The TEER value of the treated cells remained unchanged from those prior to the treatment (799.7–937.5 Ω·cm^2^). Still, in both treatments, the marked reduction in Caco-2 cell viability was observed at supernatant concentrations of 25% (*v*/*v*) and 50% (*v*/*v*) (*p* < 0.05). As for the supernatant from the enzyme mixture (Figure 3e), Caco-2 cell viability was slightly decreased at the concentrations of 5% (*v*/*v*) and 50% (*v*/*v*) (*p* < 0.05) compared to those at control concentration, i.e., 0% (*v*/*v*), but viability remained above 75%. Additionally, the TEER values of enzyme mixtures (5–50% *v*/*v*) and all treatment groups at 10% *v*/*v* showed no significant changes from baseline levels. This indicates that the enzyme mixture used during the in vitro protein digestion did not exert any confounding effects on the test. It is worth noting that in the positive control experimental set, meaning when the Caco-2 cells were treated with 0.1% Triton-X 100, cell viability was 1.3%.

**Figure 4:**
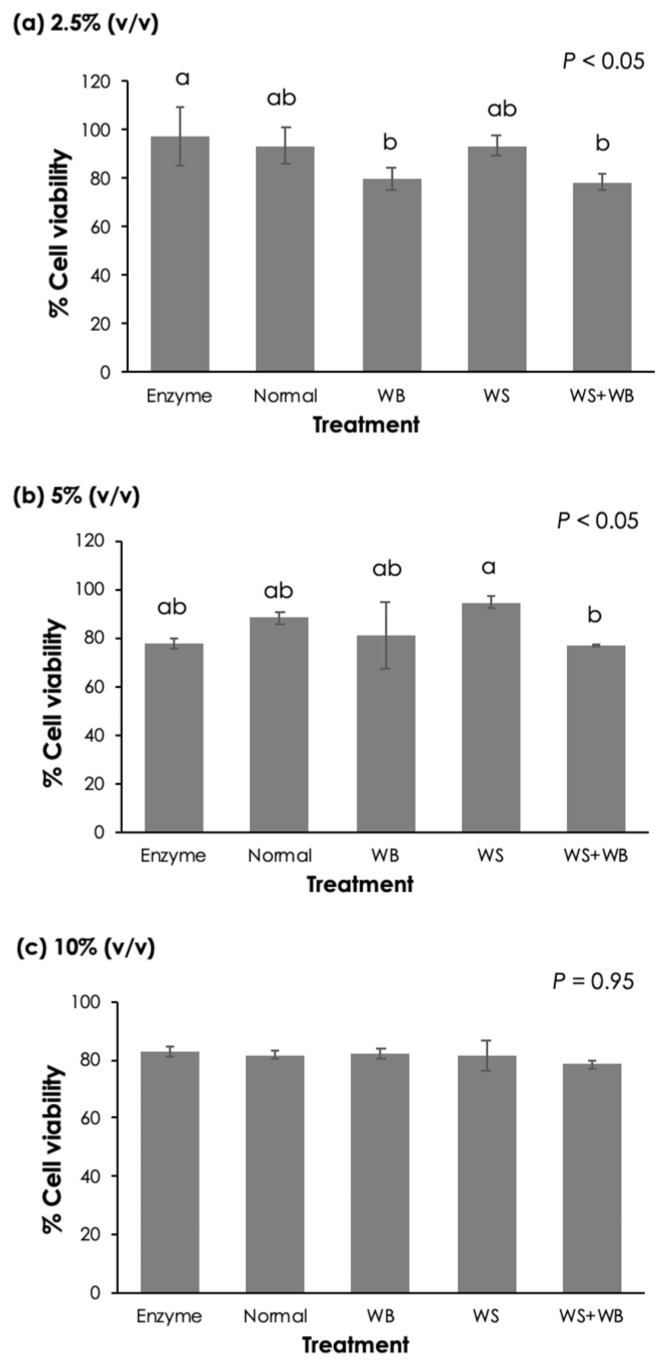
Effects of different growth-related myopathies (i.e., normal, white striping (WS), wooden breast (WB), and WS + WB) among chicken breasts on Caco-2 cell viability. The cells were treated with (**a**) 2.5% (*v*/*v*), (**b**) 5.0% (*v*/*v*), and (**c**) 10% (*v*/*v*), with the supernatants obtained via in vitro protein digestion. Bars and error bars represent the mean and standard deviation, respectively. Different letters above bars indicate a significant difference (*p* < 0.05).

Focusing on the effects of growth-related myopathies on cell viability (Figure 4), for the treatment with a 2.5% (*v*/*v*) concentration (Figure 4a), no significant differences were observed among the myopathic groups (*p* ≥ 0.05). However, the cells treated with WB and WS + WB samples exhibited a lower cell viability than those treated with the enzymatic mixture (*p* < 0.05). At the supernatant concentration of 5% (*v*/*v*) (Figure 4b), the average cell viability of the WS + WB samples (78.6%) was lower than that of the WS group (94.6%) (*p* < 0.05). Once the cells were treated with 10% (*v*/*v*) supernatant, all treatment groups exhibited Caco-2 cell viability ranging between 78.5% and 82.9% with no statistical differences (*p* ≥ 0.05). The results suggested that WB and WS + WB lesions might potentially exert negative impacts on Caco-2 cells to a greater extent compared with the other myopathic conditions.

### 3.3. Gene Expression Analysis

**Figure 5:**
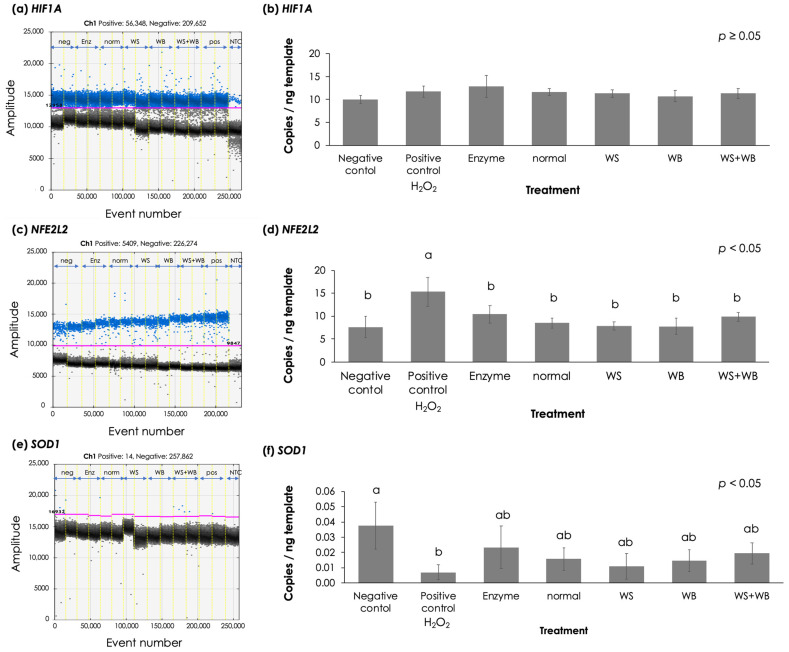
Absolute transcript quantification in Caco-2 cells using a droplet digital polymerase chain reaction. Effects of different growth-related myopathies (i.e., normal, white striping (WS), wooden nreast (WB), and WS + WB) were analyzed on three oxidative-stress response genes, including (**a**,**b**) *HIF1A*, (**c**,**d**) *NFE2L2*, and (**e**,**f**) *SOD1*. (**a**,**c**,**e**) One-dimensional scatter plots illustrate the positive droplets (blue dots above pink lines), containing target amplicons, and the negative droplets (dark dots below pink lines) without any amplicons. (**b**,**d**,**e**) Bar graphs depict the average transcript abundance (copies in 20-μL PCR reaction per ng template). Bars and error bars represent the mean and standard error, respectively. Different letters above bars indicate a significant difference (*p* < 0.05).

To further elucidate the biological response of Caco-2 cells exposed to the P5 fractions, absolute transcript abundances of genes associated with the primary oxidative stress response were examined using ddPCR. In this study, the cells were exposed only to the 0.2 m filtrates at the concentration of 2.5% (*v*/*v*). This was due to the observable decreased cell viability in the WB and WS + WB groups compared with the enzyme mixture group. Absolute transcript abundances are depicted in Figure 5. No significant differences were observed for *HIF1A* (*p* ≥ 0.05). On the other hand, significant differences in transcript abundance were detected for *NFE2L2* and *SOD1* (*p* < 0.05). Considering *NFE2L2*, its abundance in the cells exposed to H_2_O_2_ was significantly higher than those exposed to P5 fractions and the negative control (*p* < 0.05). However, when comparing among those cells exposed to P5 fractions of the enzyme mixture and the meat hydrolysates, the *NFE2L2* levels were not significantly different across the treatments (*p* ≥ 0.05). As for *SOD1*, its abundance in the cells exposed to H_2_O_2_ was significantly lower than that of the negative control (*p* < 0.05). Its expression levels in the cells exposed to P5 fractions, however, did not differ from the positive and negative controls (*p* ≥ 0.05).

## 4. Discussion

Among land animals, chicken has been projected as an essential protein source for human consumption within the next decades [45]. However, natural resources, including land, water, and clean air, will be inadequate for growing livestock. Therefore, it is crucial to determine how best to maximize the quality of the chicken meat produced in the future. However, today, most chicken meat, particularly the breast portion, exhibits at least one of the growth-related myopathies. Apart from an extensive investigation regarding the impacts of the myopathies on meat technological properties [8,46,47,48,49,50,51], as well as the etiological identification [7,13,52,53,54,55,56], little is known about the influence of abnormal meat on human health. The ultimate goal of this study was to obtain a better understanding on this aspect.

Initially, it was hypothesized that the oxidized proteins within the affected birds would disrupt the interaction between the proteases and the target binding sites. However, our recent studies demonstrated the opposite [20,21]. The cooked abnormal meat showed a greater in vitro degree of protein hydrolysis compared with the cooked normal meat. The previous observation suggested that muscle fiber fragmentation due to the growth-related myopathies [21] would facilitate enzymatic actions. In this study, the peptidomic data were in accordance with the previous reports. The mass distributions of the in vitro digested samples affected by the growth-related myopathies slightly differed, tending towards a smaller mass, compared to the cooked normal meat. The numbers of compounds with a mass < 1000 Da, corresponding with free amino acids and di- and tripeptides [43], were slightly higher in the abnormal samples. A recent study by Chang et al. [57] demonstrated that low-MW whey protein hydrolysate, containing peptides with MW values ranging between 259 to 1000 Da, showed an increased intestinal permeability compared with whey protein concentrate (MW range of 5800 to 186,000 Da). In this regard, the current findings might imply that the digested compounds of the abnormal meat would potentially be absorbed in the small intestine at a greater extent compared to normal samples. Differential peptides profiles among normal, WB, WS, and WS + WB samples were observed, suggesting that upon development of growth-related myopathies, the associated proteins of those peptides (Supplementary Figure S1) might be subjected to different degrees of proteolysis. Another plausible speculation concerning this aspect is that those proteins were highly expressed in the particular myopathic conditions. The latter case was similar, with a different protein composition in a different muscle type [58]. An investigation into protein side chain chemical modification due to growth-related myopathies is underway to comprehend those aspects.

Given that the WB meat showed further muscle degradation [21,42], this led to a hypothesis that the pathological conditions within the WB samples would be severe compared to the normal and WS samples. Such conditions might induce adverse phenomena in the human gut. In this study, 0.2 μm filtrates of P5 fractions, corresponding to the gastrointestinal protein hydrolysate, were applied to human epithelial Caco-2 cells. The results indicated the dose-dependent effects of all samples, with or without growth-related myopathies, on Caco-2 cell viability. The findings aligned well with the fundamental toxicological concept underlying the dose–response relationship [59]. Focusing on the filtrate concertation of 2.5% (*v*/*v*), despite there being no significant differences in cell viability among normal and all abnormal samples, the WB and WS + WB samples significant lowered the cell viability compared to that of the enzyme mixture. The results suggested that the development of WB lesions appeared to exert a stronger effect on Caco-2 cells.

To further elucidate the molecular response when the Caco-2 cells were exposed to the P5 fractions, the transcript abundance of oxidative stress response genes was determined. Based on the current ddPCR results, *HIF1A* transcript abundance was not different among the samples. Because HIF1A is a master responsive regulator towards limited hypoxic condition, the present findings suggested that oxygen availability was comparable among the treated cells (Figure 5a). Hence, the observable deleterious impacts of WB and WS + WB samples might not be relevant with limited oxygen availability in the cells. As for *NFE2L2*, this gene encodes *NFE2L2* transcription factor regulating molecular responses against high level of ROS. By binding to the antioxidant response elements, *NFE2L2* upregulates transcription of various antioxidant genes [26]. Herein, the Caco-2 cells treated with H_2_O_2_ exhibited an increased *NFE2L2* abundance (Figure 5b) over the negative control and other treatments. The results demonstrated an activation of *NFE2L2* in the Caco-2 cells upon exposure to H_2_O_2_. The current results aligned well with a recent study of Shao et al. [60] which reported increased *NFE2L2* in H_2_O_2_-induced oxidative stress in goat epithelial cells. On the other hand, no significant differences in *NFE2L2* transcript abundance were detected among the other treatments, suggesting no marked signaling of oxidative stress upon exposure to P5 fractions of the chickens with or without growth-related myopathies. Regarding *SOD1*, the enzyme encoded by this gene is widely recognized as a frontline responsive signal against ROS [61]. Hence, the lower *SOD1* transcript abundance in the cells exposed to 5 mM H_2_O_2_ compared with the negative control was initially unexpected. Previously, Baldelli et al. [62], addressed an interaction between *SOD1* and neuronal nitric oxide synthase (nNOS) in preventing cellular nitrosative damages. They reported an *SOD1* downregulation when nitric oxide was overly produced within the cells. Given that our positive Caco-2 cells control were exposed to 5 mM H_2_O_2_, one could speculate that an exposure to H_2_O_2_ might induce a transcriptional activation of NOS [63], leading to the suppression of *SOD1* [62]. The current results suggested a complex interplay among biological signaling pathways in mediating oxidative stress within the Caco-2 cells. Nonetheless, the cells treated with the 0.2 μm filtrates of P5 fractions showed intermediate *SOD1* levels between the positive and negative control experimental sets. Thus, it could be speculated that exposure to the 0.2 μm filtrates of P5 fractions induced certain levels of stress to the cells. However, the harmful effects of P5 factions were at a lesser extent than an exposure to 5 mM H_2_O_2_. It is worth noting that the *SOD1* transcriptional levels were relatively low compared to the other target genes. In addition, no significant differences in *SOD1* abundance were detected between the normal and abnormal samples. Based on the current findings, it is reasonable to consider that the cooked chicken meat with growth-related myopathies did not exert significant oxidative stress at a molecular level on the human epithelial Caco-2 cells.

## 5. Conclusions

In conclusion, the current study addressed the differential peptide profiles among in vitro digested cooked chicken meat with different growth-related myopathies, i.e., normal WS, WB, and WS + WB. The development of such myopathies showed no significant detrimental effects on Caco-2 cell (*p* ≥ 0.05). Nonetheless, WB hydrolysates significantly lowered Caco-2 cell viability when the concentration of the hydrolysate was at 2.5% (*v*/*v*). As the concentration increased, no significant differences in the human epithelial cell viability were detected among the meat hydrolysates. No significant differences in absolute transcript abundances of primary oxidative stress response genes, including *HIF1A*, *NFE2L2*, and *SOD1*, were found in the tested Caco-2 cells among the normal and abnormal samples. Overall, our current findings demonstrated that cooked chicken meat with growth-related myopathies did not exert significant oxidative stress at molecular levels to the human epithelial Caco-2 cells after a single exposure. However, an in-depth investigation, including the effect of long-term exposure, remains to be explored to better comprehend the effects of consuming chicken meat with growth-related myopathies on health aspects.

## Figures and Tables

**Table 1:** Primers for gene expression analysis in Caco-2 cells.

Accession NO.	Gene ID	Sequence (5′-3′)	Amplicon Size (bp)	Annealing Temperature (°C)	Template Used (ng)
AF208487.1	*HIF1A*	F: TCACCTGAGCCTAATAGTCC	121	55	500
		R: ATGGGTTCTTTGCTTCTGTG			
KJ891696.1	*NFE2L2*	F: CACATCCAGTCAGAAACCAG	143	60	25
		R: GCCGAAGAAACCTCATTGTC			
NM_000454.5	*SOD1*	F: TCTGTGATCTCACTCTCAGG	168	60	100
		R: AGGGAATGTTTATTGGGCGA			

**Table 2:** Relative quantity (as a percentage) of <1000 Da peptides to total released peptides in the in vitro digested chicken meat samples with different growth-related myopathies.

Sample	% Mass Distribution
Normal	85.13
WB	86.64
WS	84.43
WS + WB	83.65

## Data Availability

The MS/MS raw data and analysis files have been deposited in the ProteomeXchange via the jPOST partner repository with the dataset identifiers JPST003455 and PXD057480. Other data presented in this study are available on request from the corresponding author.

## Supplementary Materials

The following supporting information can be downloaded at: https://www.mdpi.com/article/10.3390/foods13244042/s1, Figure S1–S5: Peptidomics reveals differential peptides (FDR < 0.05) in in vitro digested cooked chicken breasts with or without growth-related myopathies. Their respective proteins and protein biological roles are included. Figure S1: Heat map depicts highly abundant peptides observed in cooked normal samples; Figure S2: Heat map depicts highly abundant peptides observed in cooked samples affected with Wooden Breast (WB) condition; Figure S3: Heat map depicts highly abundant peptides observed in cooked samples affected with White Striping (WS) condition. Figure S4: Heat map depicts highly abundant peptides observed in cooked samples affected with both White Striping (WS) and Wooden Breast (WB) conditions, i.e., WS + WB. Figure S5: Heat map depicts highly abundant peptides observed in cooked normal and WS samples.
