# Supplementary material for: Influences of Growth-Related Myopathies on Peptide Patterns of In Vitro Digested Cooked Chicken Breast and Stress-Related Responses in an Intestinal Caco-2 Cell Model
Source: Foods. 2024 Dec 14;13(24):4042. doi: 10.3390/foods13244042 (PMC11727595; doi:10.3390/foods13244042)
Supplement: Supplementary file 1 [file foods-13-04042-s001.zip › foods-3327380-supplementary.pdf]

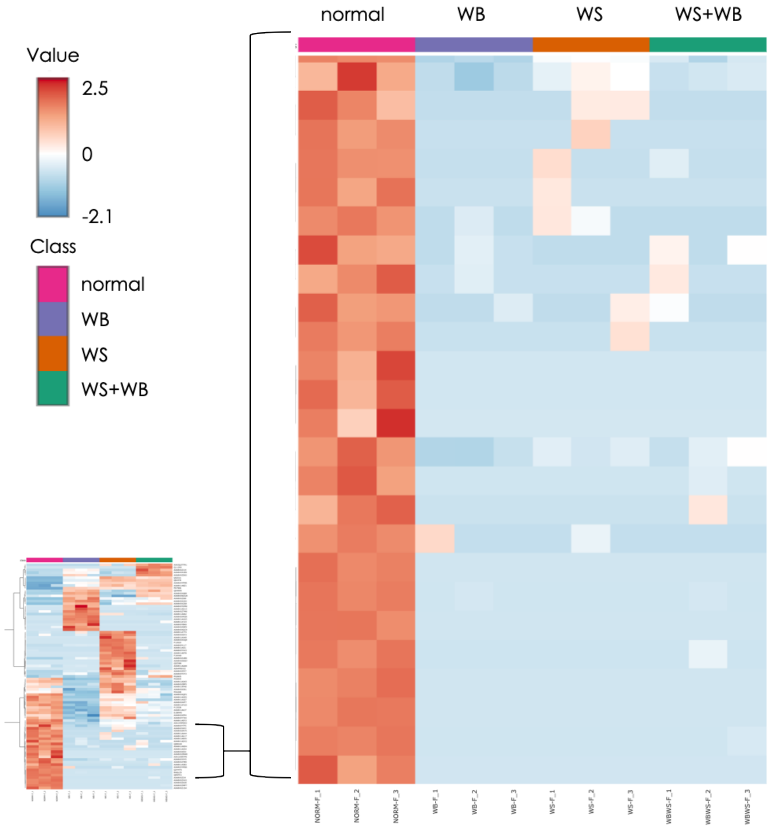

**Protein ID**	**FDR**	**Biological roles**
COL4A6	0.015	extracellular matrix structural constituent
MROH1	0.018	lysosomal regulator
FKBP10	0.016	calcium ion binding [GO:0005509]; peptidyl-prolyl cis-trans isomerase activity [GO:0003755]
Keratin	0.009	structural molecule activity [GO:0005198]
MYO1	0.004	cytoskeletal motor activity
RPRD2	0.003	RNA polymerase II C-terminal domain binding [GO:0099122]
CRLF3	0.013	cell-cell communication
BTC	0.009	epidermal growth factor family
ZNF618	0.020	protein dimerization activity [GO:0046983]
HYAL1	0.004	hyaluronoglucosaminidase activity [GO:0004415]]
Ig-like dom	0.001	cell-cell recognition, cell-surface receptors, muscle structure, immune system
PEX5L	0.001	regulator of lysosomal gene expression and autophagy
SHC1	0.029	ephrin receptor binding [GO:0046875]; epidermal growth factor binding
LOC101749377	0.008	RNA binding [GO:0003723]
DOCK7	< 0.001	guanyl-nucleotide exchange factor activity [GO:0005085]
RPESL	0.010	SMB domain-containing protein
SLC38A9	0.015	neutral amino acid transporter
CD200R1B	< 0.001	signaling receptor activity [GO:0038023]
TCF21	< 0.001	transcription factor
MHC class I antigen	< 0.001	cell-mediated immunity
NTN4L	< 0.001	secreted guidance factors
Uncharacterized	0.0298	cell-mediated immunity
Paired box 1	< 0.001	DNA binding [GO:0003677]
AACS	< 0.001	acetoacetate-CoA ligase activity [GO:0030729]; ATP binding [GO:0005524]
NOX5	< 0.001	calcium ion binding [GO:0005509]; oxidoreductase activity [GO:0016491]

Supplementary Figure S1: Peptidomics reveals differential peptides (FDR < 0.05) in in vitro digested cooked chicken breasts with or without growth-related myopathies. Heat map depicts highly abundant peptides observed in cooked normal samples. Their respective proteins and protein biological roles are included.

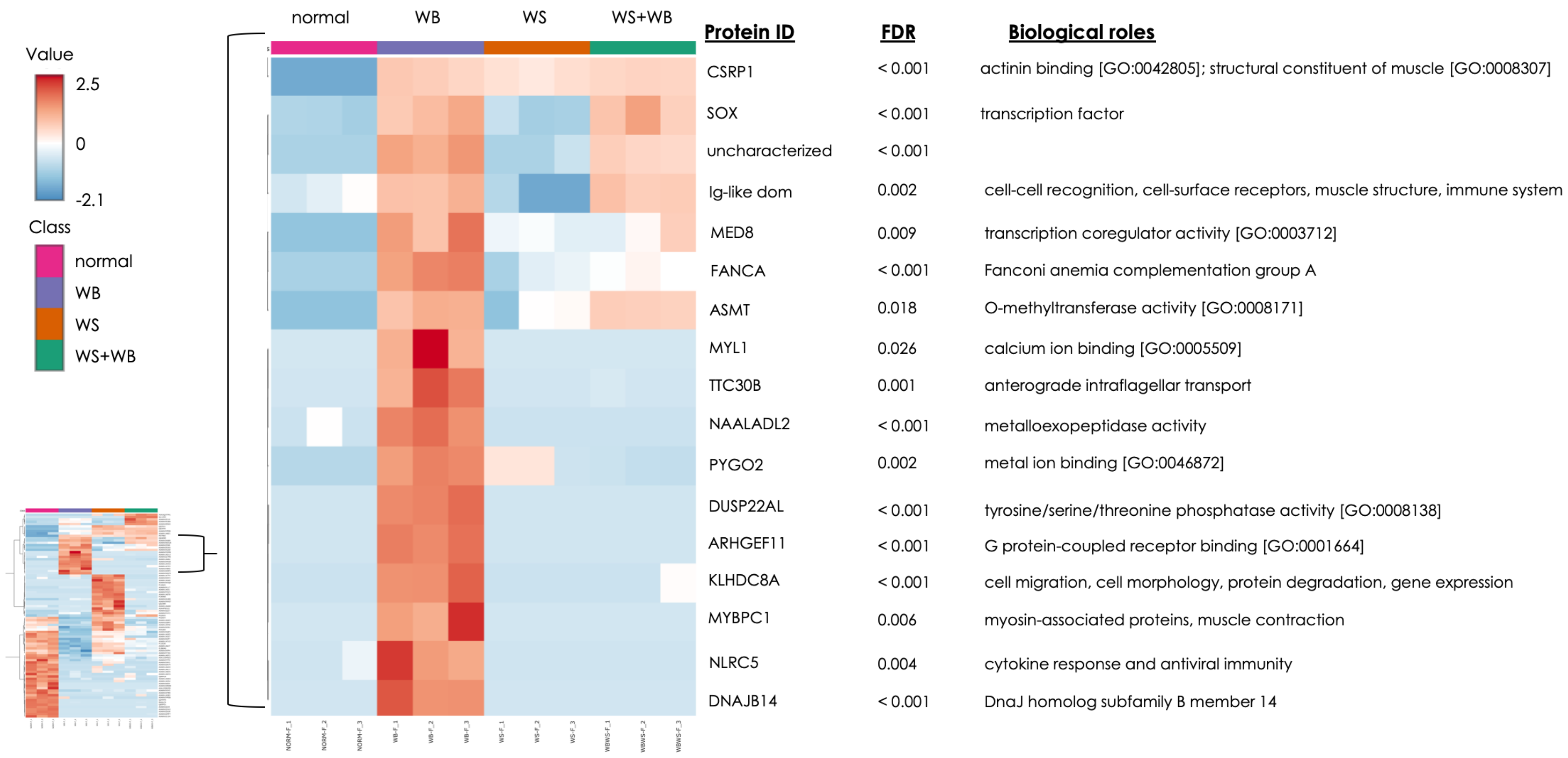

Supplementary Figure S2: Peptidomics reveals differential peptides (FDR < 0.05) in in vitro digested cooked chicken breasts with or without growth-related myopathies. Heat map depicts highly abundant peptides observed in cooked samples affected with Wooden Breast (WB) condition. Their respective proteins and protein biological roles are included.

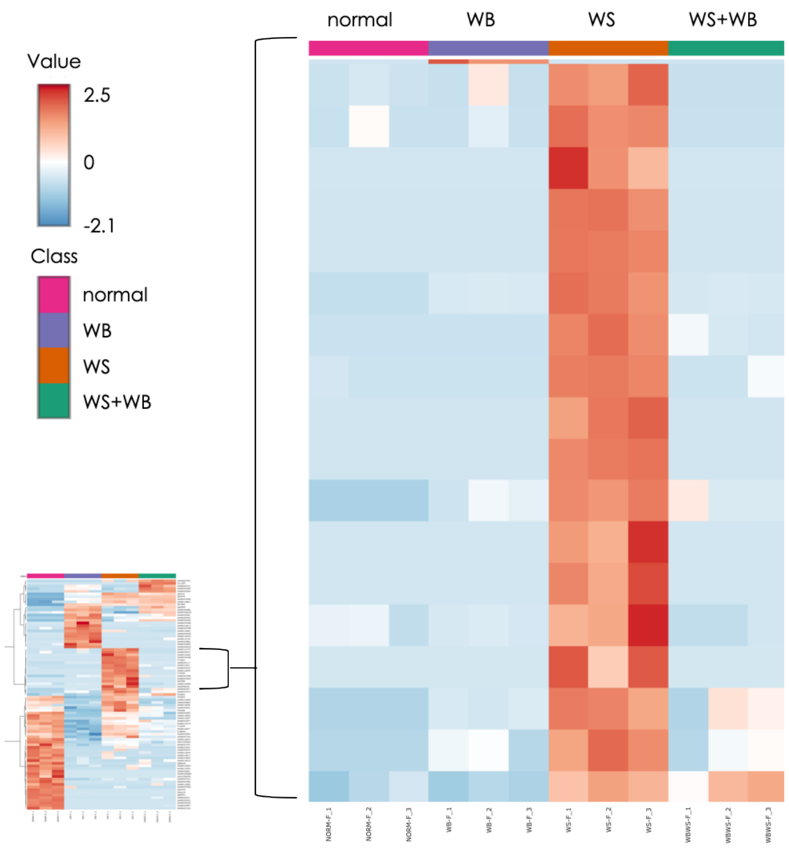

Protein ID	FDR	Biological roles
KCNH2	0.005	voltage-gated potassium channel activity
CPA2	< 0.001	metallocarboxypeptidase activity [GO:0004181]; zinc ion binding [GO:0008270]
Mucin-5AC	0.008	gel-forming glycoprotein of gastric and respiratory tract
COQ6	< 0.001	oxidoreductase activity, ubiquinone biosynthesis monooxygenase
TNNT3	< 0.001	muscle structure, muscle contraction
SLC16A10	< 0.001	transmembrane transporter activity [GO:0022857]
ST14	< 0.001	serine-type endopeptidase activity [GO:0004252]
FRMPD3	< 0.001	regulation of G protein-coupled receptor signaling pathway
TOMM40L	< 0.001	protein transmembrane transporter activity
EEF1B2	< 0.001	guanyl-nucleotide exchange factor activity
Kinesin-like protein	< 0.001	ATP binding [GO:0005524]; microtubule motor activity [GO:0003777]
QKI	0.005	RNA binding [GO:0003723]
PIK3R5	< 0.001	1-phosphatidylinositol-3-kinase regulator activity
ASH1L	0.019	chromatin binding [GO:0003682]; DNA binding [GO:0003677]
TRHR3	0.015	thyrotropin-releasing hormone receptor activity [GO:0004997]
SKIVL2	0.013	assists the RNA-degrading exosome during the mRNA decay and RNA quality-control
APS2	0.011	anterograde synaptic vesicle transport and vesicle-mediated transport
MYL3	0.012	muscle structural formation and maintenance, muscle function

Supplementary Figure S3: Peptidomics reveals differential peptides (FDR < 0.05) in in vitro digested cooked chicken breasts with or without growth-related myopathies. Heat map depicts highly abundant peptides observed in cooked samples affected with White Striping (WS) condition. Their respective proteins and protein biological roles are included.

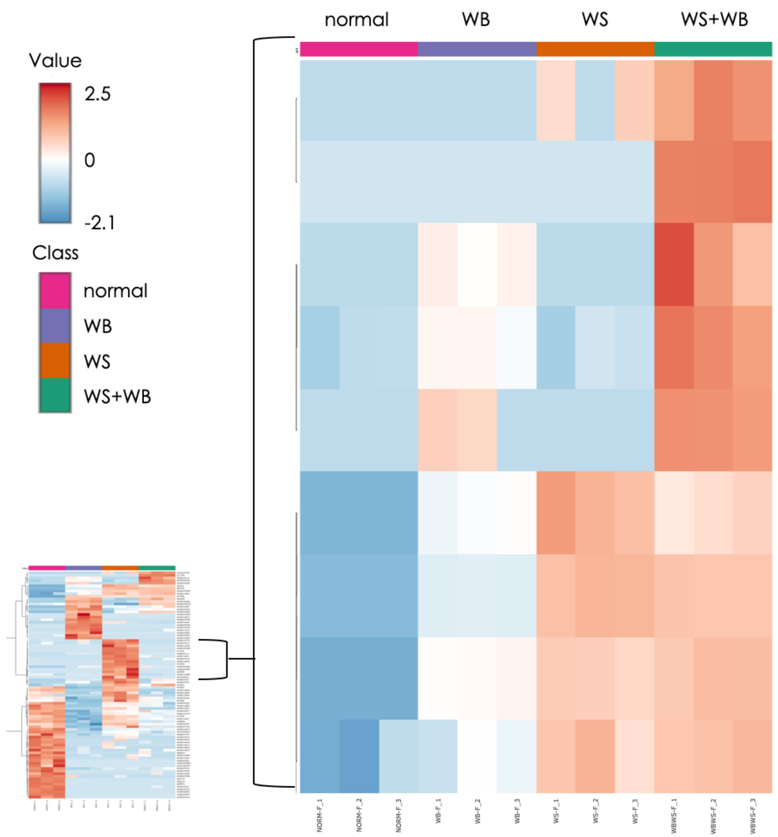

Protein ID	FDR	Biological roles
CRYAB	0.022	molecular chaperone for misfolded proteins to prevent protein aggregation
ST3GAL1	< 0.001	ganglioside biosynthetic process
Prosaposin	0.004	lysosomal degradation of sphingolipids
STOX2	< 0.001	DNA-binding transcription factor activity
CNP	0.020	2',3'-cyclic-nucleotide 3'-phosphodiesterase activity [GO:0004113]; RNA binding
Uncharacterized	< 0.001	
FUS/TLS	< 0.001	RNA processing, RNA transcription; miRNA processing
CNIH3	< 0.001	regulates gating properties of AMPA-selective glutamate receptors
SPCS1	0.011	component of the microsomal signal peptidase complex; cleavage of signal peptides

Supplementary Figure S4: Peptidomics reveals differential peptides (FDR < 0.05) in in vitro digested cooked chicken breasts with or without growth-related myopathies. Heat map depicts highly abundant peptides observed in cooked samples affected with both White Striping (WS) and Wooden Breast (WB) conditions, i.e., WS+WB. Their respective proteins and protein biological roles are included.

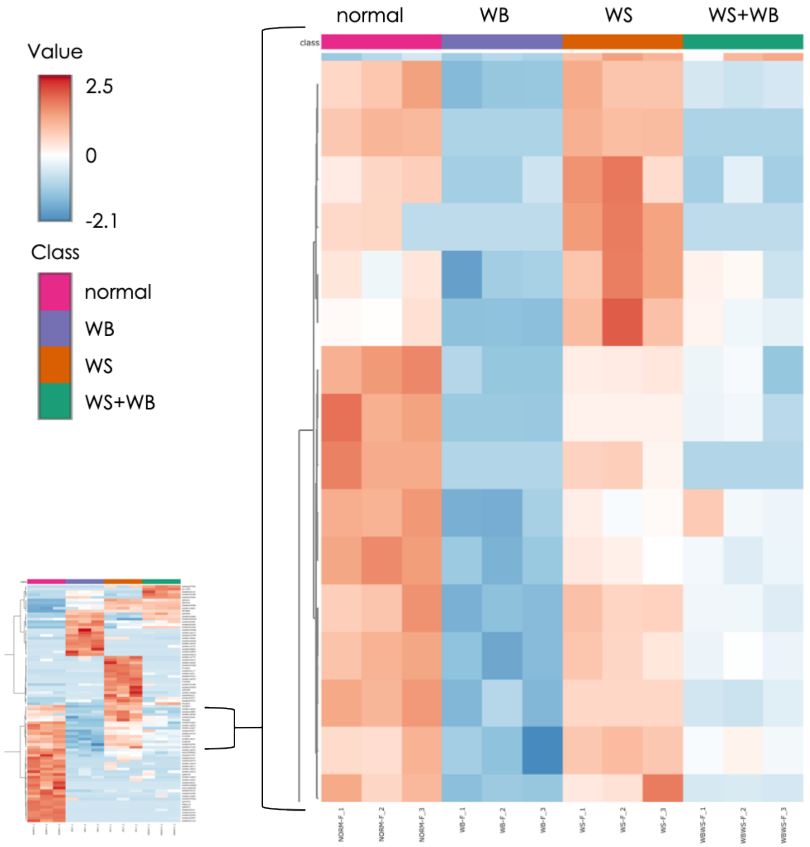

**Protein ID**	**FDR**	**Biological roles**
MYL1	< 0.001	calcium ion binding [GO:0005509]
NASP	< 0.001	histone transportation into the nucleus of dividing cells
PECR	0.029	reduction of trans-2-enoyl-CoAs; fatty acid elongation
MYH1F	0.020	muscle structure; muscle contraction; actin binding
LOC107050559	0.022	fructose-bisphosphate aldolase activity
TPM1	0.009	muscle structure; muscle contraction
SGK1	0.008	ATP binding [GO:0005524]; protein serine/threonine kinase activity [GO:0004674]
RBM8A	0.001	RNA binding [GO:0003723]
SCNM1	< 0.001	metal ion binding [GO:0046872]; sodium channel
ALDOA	0.006	fructose-bisphosphate aldolase activity
Uncharacterized	< 0.001	metal ion binding [GO:0046872]
MYH	< 0.001	skeletal muscle structure; thick filament component; muscle contraction
ELN	0.002	extracellular matrix structural constituent
STON1	< 0.001	cargo adaptor activity [GO:0140312]
WAC	0.007	regulate gene transcription; DNA damage control; autophagy
AKNAD1	0.027	protein domain on transcription factor

Supplementary Figure S5: Peptidomics reveals differential peptides (FDR < 0.05) in in vitro digested cooked chicken breasts with or without growth-related myopathies. Heat map depicts highly abundant peptides observed in cooked normal and WS samples. Their respective proteins and protein biological roles are included.
